# Factors Impacting the Uptake of Research into Dietary Sodium Reduction Policies in Five Latin American Countries: A Qualitative Study

**DOI:** 10.1016/j.cdnut.2023.100073

**Published:** 2023-04-01

**Authors:** Janice Padilla-Moseley, Bridve Sivakumar, Nadia Flexner, Ruben Grajeda, Brenda Gamble, Adriana Blanco-Metzler, JoAnne Arcand

**Affiliations:** 1 Faculty of Health Sciences, Ontario Tech University, Oshawa ON, Canada; 2 Department of Nutritional Sciences, University of Toronto, 1 King’s College Circle, Toronto, Ontario, Canada; 3 Department of Non-Communicable Diseases and Mental Health, Pan American Health Organization, Washington, DC, United States of America; 4 Costa Rican Institute of Research and Teaching in Nutrition and Health, Tres Ríos, Costa Rica Costa Rica

**Keywords:** diet, nutrition, public health policy, health promotion, sodium, research implementation, global science countries

## Abstract

**Background:**

Diets high in sodium are a risk factor for cardiovascular disease (CVD). Latin American countries (LAC) consume more than double the recommended sodium levels. Research uptake in dietary sodium reduction policies has been inconsistent in LAC, and the factors impacting research uptake are largely unknown. This study aimed to describe the barriers and facilitators to the uptake of research into sodium reduction policies from a funded research consortium with 5 LAC (Argentina, Brazil, Costa Rica, Paraguay, and Peru).

**Methods:**

A qualitative case study included 5 researchers and 4 Ministry of Health officers from the funded consortium. Dimensions from Trostle’s framework of actors, content, context, and process and relative advantages from the Diffusion of Innovation informed the semi-structured interview guide and analysis. One-on-one interviews were completed from November 2019 to January 2020. The participants validated transcripts, coded, and analyzed using NVivo software.

**Results:**

Key barriers to policy advancements included *1*) conflicts of interest from the food industry and some government actors; *2*) government turnover resulting in policy and personnel changes; *3*) a lack of human and financial resources; and *4*) and communication gaps among key actors. Key facilitators to policy advancement included: *1*) the content and quality of health economic, food supply, and qualitative data; *2*) support, technical assistance, and alliances with the government, non-governmental organizations, and international experts; and *3*) researchers enhanced skillsets facilitated with communication and dissemination with policymakers.

**Conclusion:**

Researchers and policymakers are faced with several barriers and facilitators on research uptake in policies and programs in LAC; these factors should be addressed and leveraged to advance sodium reduction policy development. Future LAC studies can draw from the insights and lessons learned from this case study and apply the results to future efforts on policy nutrition to promote healthy eating and reduce CVD risk.

## Background

Globally, excess dietary sodium is responsible for approximately 3 million deaths annually [1]. High sodium intake increases blood pressure and risk of hypertension, CVD, stroke, cancer, and kidney disease [2]. In low and middle-income countries (LMIC), diets high in sodium are responsible for 70 deaths per 100,000 individuals [1]. This risk is particularly high in Latin American countries (LAC) where up to 40% of adults have hypertension, which rates among the highest in the world [3, 4]. Additionally, sodium intakes in LAC are often more than double the recommended amount of 2000 mg/day of sodium (5 g/day of salt intake) [5]. For example, the mean population sodium intake in Argentina is 4480 mg/day, in Brazil is 4720 mg/day, in Costa Rica is 4600 mg/day, in Paraguay is 5480 mg/day, and in Peru is 3880 mg/day [[6], [7], [8], [9], [10], [11], [12]].

The World Health Organization (WHO) has encouraged member states to reduce population sodium intake by 30% by 2025, as one of the 9 voluntary global targets for noncommunicable disease (NCD) prevention [13]. Moreover, the WHO recognized reducing dietary sodium as a “Best Buy” public health intervention to reduce NCDs, as it is highly cost-effective and relatively feasible to implement [[14], [15], [16]]. For instance, in Argentina, sodium reduction policies would avert 55,000 total deaths, 27,000 stroke-related deaths, 16,000 coronary artery disease, 5000 stroke, and 38,000 myocardial infarction cases over 10 years [16]. In Brazil, reducing the population sodium intake to 2000 mg/day in adults would lead to a projected USD 102 million cost saving from reduced CVD hospitalization [17]. In Costa Rica, reducing population intakes to 2000 mg sodium/person/day would lead to the prevention of 13% of deaths from CVD if the country’s national plan for the reduction of salt and sodium is met by 2021 [18]. National population-wide sodium reduction policy interventions have been planned or developed in at least 75 countries [19], which commonly include food reformulation, consumer education, front-of-pack labeling, and taxing salt and foods high in sodium [20]. Approximately 60% of these initiatives involved regulatory approaches [19], such as food labeling and taxation; however, most countries have taken voluntary approaches to set sodium reduction targets in processed and packaged food. Despite an increase in sodium reduction policies and programs, only a few countries have reported reduced mean sodium intake [19], and to our knowledge, no LAC have met the WHO’s global sodium target, suggesting that the implementation of more effective and progressive policies may be needed [19].

Although high-quality research is critical to guiding public health policy [[21], [22], [23]], evidence-informed policymaking is complex and unique, especially in LMIC [24]. A lack of available evidence, time constraints, policymaker skills in interpreting research and transferring knowledge, financial resources, private sector influence (e.g., funding and/or lobbying), and competing political priorities were identified as barriers in LMIC to the uptake of research into policies [[25], [26], [27]]. In LMIC, the complexities of policy making are exacerbated [25] by resource constraints and unpredictable political climates [21]. There are only a few known facilitators such as access to relevant research and engagement with stakeholders at the onset of the research cycle [25, 27]. Likewise, few studies have identified the factors influencing the use of research to inform sodium reduction policies in LMIC and LAC, where contextual factors may present unique challenges compared with other countries and regions. Therefore, this study aimed to describe the barriers and facilitators to the uptake of dietary sodium reduction research in public health policies in 5 LMIC in Latin America. To achieve this aim, activities carried out by a LAC dietary sodium research consortium were examined. The consortium consisted of several health and academic institutions and organizations from Argentina, Brazil, Costa Rica, Paraguay, and Peru and was funded by the International Development Research Centre (IDRC). The core objective of the consortium was to generate and mobilize sodium reduction research that included food supply, social marketing, and health as well as economic impact analyses, to inform sodium policy in each of the funded countries. This funding led to several dietary sodium policy and program changes in the funded countries, as highlighted in a program evaluation report [28]. This served as an ideal case to examine the barriers and facilitators to dietary sodium research uptake in sodium reduction policies and programs, which was explored in the current study.

## Methods

### Research design

In this study, a constructivist approach was used to describe how research evidence is adopted into policies on dietary sodium reduction in LAC through a single qualitative case study. Constructivism is focused on understanding the historical and cultural contexts where people live and work while using multiple perspectives to illuminate the research topic. In qualitative case studies, a constructivist approach uses numerous perspectives and different meanings that are transactional where the research team has a personal interaction with the case. Some research team members (JA, JPM, NF, RG, ABM) had a pre-existing relationship with the public health researchers who were the participants in this study, which established an authentic connection and rapport during the research process. Data collection took place between November 2019 and February 2020 via one-on-one interviews with key public health researchers and the Ministry of Health (MOH) officers, which was accompanied by other sources of data, as described below. A qualitative case study methodological approach allowed us to explore the multiple cultural and contextual lenses, from each country that participated in the consortium, which may impact the use of research outputs produced from a funded research program [29].

#### Case

This case was a research project funded by the IDRC from September 2016 to March 2020 (IDRC 18167). The IDRC is a crown corporation of the Canadian government with a mandate to provide resources for research knowledge advancements in resource-limited regions. The case included a multi-country Latin American research consortium consisting of researchers and knowledge users from Argentina, Brazil, Costa Rica, Paraguay, and Peru (referred to as the “funded countries”). The consortium countries, led by the Costa Rican Institute of Research and Teaching in Nutrition and Health (INCIENSA) and with technical support from the University of Toronto and Ontario Tech University, received funding to conduct and disseminate research to inform sodium reduction policies and programs in each country. The funded countries are the units of analysis for this case.

#### Study participants and recruitment

A purposive criterion sampling was utilized to recruit the 5 public health researchers, one from each funded country, who received funding to carry out the research activities under the consortium. These researchers provided cultural (e.g., social standards, experiences, beliefs, and traditions) and contextual (e.g. external situational) information to inform the discussion. Using a snowball sampling approach, the researchers facilitated the recruitment of 5 MOH officers from each funded country. A MOH officer was eligible to participate if they had a leadership role in NCD development and/or implementation in the 5 funded countries. Eligible participants were invited to participate in the study via e-mail invitation. Due to scheduling conflicts with the MOH officer from Brazil, only 4 MOH officers were included in the study.

#### Data collection

Information sources included one-on-one interviews, the interviewer’s reflective memos, and a policy map developed by the Pan American Health Organization (PAHO) that cataloged sodium reduction policies in each country [30]. In addition, 45- to 60-minute semi-structured interviews took place via videoconference. Interviewers recorded reflective memos on ideas, insights, observations, and a self-critique of performance after the interview [31, 32].

Two separate semi-structured interview guides were created for each participant pool: the researchers (20 questions) and the MOH officers (16 questions). The 2 interview guides had variations in the questions to adjust the language according to the type of respondent. Two frameworks were selected a priori to inform the interview guide questions that explored developments and changes in dietary sodium reduction policies in the countries, which occurred during the funding period. In addition, the interview guide inquired about the factors related to the uptake of research outputs generated from the funded research consortium into dietary sodium reduction policies. The 4 dimensions of actors, content, context, and process that were used in the study by Trostle et al. [33] were selected as the main framework to inform the interview guide and data analysis. This allowed for the exploration of internal (e.g., government, scientists) and external (e.g., industry, civil society) influences on research uptake, as used in previous studies. In addition, the relative advantage domain from the Diffusion of Innovation (DOI), a theory commonly used to examine how research has been adopted within organizations, was used to describe how the research innovations generated under this project are better than the existing research data, if available. In addition, questions about the policy map for each country were embedded in the interview guide, providing a basis to explore the country-specific sodium-policy context and the changes in dietary sodium-related policies, programs, interventions, and regulations that occurred during the grant period. Supplemental Table 1 is a policy map of the sodium reduction policies that were revised, updated, or newly developed by the funded countries over the funding period. Using the policy maps as a guide, participants were asked to what extent the IDRC research evidence influenced policies and programs in the funded countries. Participants received an interview guide (available in Spanish and English) and a country-specific policy map before the interview. The guides were validated among the research team members (JPM, JA, BG) to ensure clarity of questions, accurate choice of words, appropriate tone, and freedom from leading questions.

Interviews were conducted in English (JPM) and Spanish (NF). These interviews were audio-recorded with Audacity. Recordings were transcribed verbatim by 2 researchers (JPM, BS), and the transcript validation was completed through comparisons with the recorded interview. A translator transcribed the Spanish interviews and then translated the transcripts into English for analysis. All direct and indirect identifiers were removed from the transcripts when participants expressed opinions, direct quotes, or identifiable information in the quote. Study participants were identified by their professional role (e.g., public health researcher or MOH officer) and an assigned country code (country A to E) to respect confidentiality. Country names were only used when stating publicly available information about the country.

### Research team and reflexivity

The research team consisted of 2 graduate students (JPM and BS), 2 associate professors, one with expertise in sodium research (JA), policy (JA, BG) and qualitative methods (BG), as well as 3 public health professionals (NF, RG, ABM) from the PAHO and INCIENSA in Costa Rica. The research team members (JA, JPM, NF, RG, ABM) were international research collaborators who served as subject matter experts on the IDRC grant and had a pre-existing relationship with the public health researchers. This involvement established an authentic connection and rapport with the researchers [34], who became participants in this study. There was no previous relationship between the MOH officers and select members of the research team.

#### Data analysis

Interviewers conducted a post-interview debrief discussion with each participant to explore preliminary interpretations and conclusions. Before analysis, participants member checked their transcripts and confirmed that no changes were required. NVivo software (Version 12) was used for data organization and analysis. Codes and a codebook were established a priori using the DOI and Trostle’s themes. The codes were expanded with sub-codes to identify emergent themes. Two independent coders (JPM, BS) deductively coded the transcripts and applied codes to relevant segmented sentences and paragraphs on participant views and experiences, as well as direct quotes. Intercoder agreement was reached through discussion and consensus, with a third researcher (JA) used to resolve any disagreements. Data authenticity was supported by the interviewer’s reflective memos and the use of a second coder.

## Results

Nine participants consisting of 5 key public health researchers and 4 MOH officers from Argentina, Brazil, Costa Rica, Paraguay, and Peru participated (2 men, 7 women). The participants were a mix of early careers and senior researchers. MOH officers were included from each country; however, the participant from Brazil was unable to participate due to scheduling conflicts. As presented in Supplemental Table 1, the 5 countries varied in their stage of policy development. Argentina and Costa Rica had existing dietary sodium reduction policies in place. However, Paraguay and Peru were at an early phase of dietary sodium reduction policy development. The barriers and facilitators described in this section were common among most of the funded countries, regardless of their stage in policy development. Countries that were in the advanced stages identified similar factors as countries who are in the early stages of development. This study did not explore barriers and facilitators to policy implementation in general; therefore, we cannot comment on relative advantages concerning overall policy development, which would describe why some countries have made very good progress in policy implementation, whereas others are laggards. One can only assume that factors beyond the availability of research evidence influence policy and program development. The identified barriers and facilitators to dietary sodium research uptake in policy are found in Supplemental Table 2.

### Barriers to the integration of research into sodium reduction policy

#### Actors

##### Food industry

Every country noted the food industry as a barrier to research implementation into policy development such as setting sodium targets (e.g., mandatory versus voluntary). A participant from country A commented that the food industry has a good understanding of the research evidence available on the health effects of decreased sodium consumption; however, no action took place that progressed to making further reductions in sodium content levels in food products. Country A participant noted that the food industry often has conflicts of interest that hinder progress. This participant did not elaborate on the specific conflicts of interest out of fear of negative repercussions. In country C, conversations between public health officials and the food industry were historically met with lobbying and resistance. This was a similar experience when funded countries were in conversations with the government on the application of taxes on foods and beverages with high sugar content and voluntary or mandatory sodium reduction target levels in food products [19].

The food industry was viewed as a highly influential partner in dietary sodium reduction policies. Their competing priorities on their own company’s economic growth were seen to impede or delay policies related to food reformulation, including the use of research in policymaking. For instance, in country D, the food industry was concerned about the acceptability of product taste when sodium was reduced in baked goods, with a concern for the potential for reduced sales and job losses. A MOH officer from country D noted that criticisms from the food industry were: “It won't taste good,” “people won’t buy it,” and “people won’t like it.” To mitigate this concern, country D partnered with a food technology team to create food products of varying sodium levels and conducted taste tests among consumers. The tests found that incremental decreases in sodium over time would not impact consumer taste preferences or negatively impact product acceptance. In country E, one company’s major revenue source derived from commonly purchased condiments; however, these products had high concentrations of sodium. It was reported that the food industry in country E had similar concerns about sodium reduction, leading to changes in consumer buying patterns. This was a major impediment to their willingness to reduce the sodium content of condiments.

All countries also expressed concerns when working with the food industry on policy and program approaches to improve the nutritional profile of imported and domestically produced exported food products. Despite the research on the sodium content of packaged foods generated by the IDRC-funded research project, country A noted that a major barrier to effective research implementation into the sodium reduction policy was that the data will not be used as approximately half of the food products sold in the country are imported. A participant from country A noted that this was common in many LAC, especially those that were small and highlighted the importance of industry engagement (especially large exporters) across the region.

#### Process

##### Human resources

A lack of human resources reduced the use of research findings in policy development and change. Two MOH officers from countries C and D reported being overstretched with their time: “Our involvement in so many projects could have slowed down this process because no one was exclusively devoted to this project [IDRC research project], and everyone had to split their hours between several projects.” [MOH Officer, country D]. They also noted that dedicated full-time human resources were required to support sodium reduction policy work as individuals worked on multiple public health priorities: “Probably this [resource] is a big barrier. It is a team that is working on several things on non-communicable diseases that was important in this project [IDRC research project] but probably we need more people working in research.” [MOH Officer, country D]. Meanwhile, country A’s momentum on sodium reduction policies and initiatives was at risk, as several researchers in the field were at retirement age with no succession plan in place. A researcher from country A noted that a lack of continuity would compromise previous sodium reduction efforts and achievements. Challenges with human resources were also observed at the leadership level. At the start of the IDRC-funded consortium, respondents noted that the PAHO demonstrated strong support in mobilizing efforts and resources on sodium reduction policies. However, PAHO's involvement as a knowledge user in the IDRC-funded consortium was impacted by economic and human resource constraints when their institutional efforts were re-distributed among other regional public health priorities, reducing the amount of time available for sodium reduction. During the interview period, participants reported that this shift in focus compromised the work required to complete the updates to the regional sodium reduction targets. Several months later, PAHO recently published and launched updated regional sodium reduction targets using the research data acquired from this project [35].

##### Communication

Communication challenges led to a lack of research being integrated into policy development and changes, as reported by countries A, D, and E. In country A, decisions stagnated with the MOH due to untimely and unanswered communications. In countries D and E, critical information from the researchers did not flow through the proper verbal and written communication channels to decision-makers. A MOH officer from country E noted that they often felt “divorced from the private sector, from academia”. Meanwhile, a researcher from the same country acknowledged that researchers and MOH worked in silos before the IDRC-funded consortium. This researcher acknowledged that the consortium triggered a change in research communication strategies, as a result of training in knowledge translation (KT). This researcher also noted that they have now designed future projects to include “dedicated time and space” for end-of-project KT activities.

#### Context

##### Frequent changes in government

In some countries, frequent changes in the government created shifts in public policy focus, which negatively impacted the uptake of research findings in policymaking. In country C, a government changeover resulted in significant shifts in political views and focus. This changeover took longer than anticipated to replace the positions. Once the positions were filled, researchers underwent extraordinary efforts to re-engage with decision-makers and restart conversations on the sodium reduction policy.

Country E experienced a high number of turnovers at the Health Minister level. A researcher in country E found it challenging to work with government individuals who did not hold a position in the office long enough to engage in public health policies. On average, an incumbent held a position for less than a year when the usual term was 5 years. Country E’s MOH officer noted that the MOH preferred timely results to coincide with the Minister’s term in office. As such, a researcher from country E noted that the Minister wanted to observe the immediate effects of their health policies; however, public health efforts involving NCDs have a long latency period for observable impacts, noting:

“[...] they want results for this year, for example, they want to reduce...I don’t know something like five points this year because they want to give results to the population and next time they will have more votes. However, in chronic diseases, you cannot see the results very soon. But with chronic diseases, it takes decades to see results.”

In contrast, country E’s MOH officer did not perceive the high turnover rate as a barrier and noted that, “Despite the fact that we have had so many Ministers at the Ministry of Health, our goals have remained unchanged.”

### Facilitators to the integration of research into sodium reduction policy

#### Content

##### Research data were generated under the IDRC-funded consortium

The research data generated under the IDRC project were collected using robust methods. For countries A and C, the health and economic impact data acquired for sodium-reduced diets were considered a strong facilitator that generated interest from key decision-makers in the country. Country C noted that this data were used to break resistance and lobbying efforts from the food industry and initiated conversations on sodium reduction targets. A researcher from country C noted:

“…that we are planting a seed for the future and trying to build a stronger case with…so that we can evaluate. We have something we are working on...as a kind of impact on regulation. It’s a report on the regulatory impact of any kind of measure and that it must include a strong case of health and economic evidence so we are trying to build that part so we can fight that battle, so to say, and in the near future.”

A MOH officer from country A emphasized that a combination of short- or long-term data demonstrating the cost-effectiveness of sodium reduction policies will generate interest from policymakers, stating:

“You have to demonstrate, you have to have evidence, if you have the evidence and you have the data of course, that will help policymakers to make decisions and that will be credible. Because if you do not have the data, there will be no interest in the integration of the policy and programs.”

Country D, and others, also highlighted that the quantitative data related to the sodium content of the food supply impacted decision-makers. A MOH from country D noted:

“with respect to the data of our current project [IDRC project], in fact, the data we obtained is quite solid because the process was done rigorously, it was carried out properly. It was partly innovative because we used technologies, such as FLIP [data collection app and food database], that we learned and got to know here. The scientific community is interested in knowing and learning about such things. […] The data we obtained from the research we carried out during the IDRC project has been very important, it is the evidence we are going to use to reach decision-makers above us, who are the ones setting the standards in terms of policies and everything else. We also collaborate in the formulation of laws, resolutions, etc., but it is ultimately up to the top decision-maker.”

##### Qualitative research data were generated under the IDRC-funded consortium

Qualitative data were believed to be influential, alongside the quantitative data for countries D and E. The MOH in country D found the qualitative data provided insights into consumer needs and opinions related to sodium, data that did not previously exist. For instance, the qualitative data found that consumers viewed iodized salt as necessary for good health due to past public health initiatives to address goiter. Country D also noted that this data provided insights on how to connect with a hard-to-reach adult population, where changing behaviors has been historically challenging. In country E, researchers noted that the strength of the qualitative research data highlighted the root causes of people’s health practices and food purchasing patterns which were not easily identified with quantitative research methods.

#### Actors

##### Government political will

The government’s strong political will manifested through outreach efforts, commitments, and mobilization of resources to support sodium reduction policies and programs in the funded countries. This was a foundation to advance political agendas on sodium reduction in the funded countries. In Argentina, a Senator contacted researchers to initiate regulatory work on sodium reduction in food products. In Peru, the IDRC study results activated the MOH’s interest in sodium reduction for the first time. This was viewed as a significant achievement in Peru, as the government’s primary focus was directed at anemia and diabetes. Both participants in Costa Rica noted that the government deemed the IDRC-funded consortium research to have national importance and committed to sodium reduction policies and programs in the country, which supported research efforts. Additionally, the Costa Rican MOH and the CACIA, Cámara Costarricense de la Industria Alimentaria (Costa Rican Chamber of the Food Industry) renewed their alliance and shared a commitment to sodium reduction in the food supply, which included updates to the sodium targets for the packaged food supply. The government in Brazil committed to addressing NCDs regardless of the political party in charge. The MOH from Paraguay mobilized additional human and financial resources to support NCD initiatives and created a new department to support the work of the IDRC-funded research consortium.

##### Research experts

Within each funded country, the expertise of regional and international researchers was seen as influential in creating opportunities to advance research into sodium reduction policies and programs. For example, in country A, the government drew on the expertise of nutrition researchers to advise and participate in advisory boards and technical expert groups to inform sodium target levels and national strategies. The researchers generated evidence with responsibility “for translating the technical aspects [of the research] into something understandable for decision-makers” [Researcher, country B].

International researchers who supported the IDRC grant were also recognized as highly influential “to have these heroes by our side [from the University of Toronto and Ontario Tech University], and because they are also communicators and they help a lot with advocacy”, as noted by a researcher from country C. In Brazil, international researchers participated in round table discussions with the Inter-American Heart Foundation, NCD Alliance, and the IDRC. These discussions ultimately led to NCD strategies for the LA region, which supported the integration of the IDRC data into national sodium reduction policies. The international recognition and credibility of researchers in Argentina and Costa Rica also led to continuous research funding commitments from international agencies, such as the IDRC, to continue with sodium reduction research to inform policies and programs.

##### The dual role

Individuals with dual roles as researchers and MOH officers were valuable liaisons that led to strong planning and coordination of the IDRC-funded consortium research activities, as well as the use of the data in policymaking. In Brazil, the dual roles allowed researchers to be privy to insider information, which allowed them to be in a better position to hold discussions with policymakers and key stakeholders and to more effectively generate and transfer relevant, audience-specific research data.

In Paraguay, the MOH participated in social marketing research, which allowed them to directly apply the research findings to an awareness campaign, in addition to dissemination to academia and scientific organizations. In parallel, Paraguay’s MOH involvement with research activities enhanced their credibility and ability to advocate for the use of sodium reduction research with decision-makers, noting:

“Our cooperation with academia would be a facilitator because people respect researchers and scientists; they are perceived as more knowledgeable and trustworthy. If they take our results and information that will work in our favor, it will be a facilitator in all the measures we need.”

##### Non-Governmental Organizations

Many countries had a positive perception of international non-governmental organizations such as the Food and Agriculture Organization of the United Nations and PAHO. All countries perceived PAHO as positive and highly credible. As such, any endorsements of the research from PAHO were viewed as a strong facilitator of change that led to the uptake of research findings in policies. An MOH officer from Country D mentioned that:

“There is a positive perception of PAHO’s support at the Ministry of Health. People trust their opinion; they believe that if PAHO supports something, it must be reliable; it must be true. The technical support they provide is important during the entire process, mostly when we first approach academia and when we start showing scientific evidence. Thanks to their support, we can count on foreign experts who come, share their experience, and support us with what we are starting right now.”

##### International Support

Scientific evidence generation in NCD for use in policy making is highly dependent on external funds and support from international agencies such as the IDRC. All countries mentioned that the IDRC’s funding and support enabled them to generate sodium reduction research and motivated conversations in the LAC on sodium reduction targets in food products. Country B’s participant noted that if, “IDRC had not funded our project, we would not be able to start working in this field.” All participants from each funded country expressed their accolades for the IDRC and their commitment to funding research and innovation in developing regions. Country B’s public health researcher stated, “IDRC is not like any other funders, they are very interested in funding future research to monitor processed sodium levels in processed foods that may become an obstacle or barrier for us to be able to continue monitoring the policy.”

##### Media

The media was viewed as a strong vehicle to garner public interest to stimulate policy development and change. In country B, the media disseminated the funded research data, which drew attention to the high sodium levels in the food supply. Both the MOH officer and researcher from country B believed that the media was a key driver in sodium reduction regulations and that they planned to leverage the impact of the media in the future. Additionally, the media in country C was used to restart conversations on public health issues that are not permanently in the spotlight and easily overlooked, such as setting new sodium reduction targets and disseminating the monitoring results. In country E, the media was similarly viewed as a catalyst to drive changes with sodium reduction efforts, similar to how the media was previously used to stimulate changes to food policy and nutrition legislation. In country D, the media was useful in promoting communications on the social marketing plan developed under the IDRC funding.

#### Process

##### KT Training and Implementation

Activities under the IDRC-funded consortium research introduced a new way of disseminating research through formalized KT training and strategy development. Researchers noted that an integrated KT approach enabled new ways of early engagement between researchers and policymakers during the research process:

“When we have any research grants for small funded projects, we have added something that was inspired by the IDRC project...is that this communication plan…this knowledge transfer. It is something that is very important for presenting the results, making the results easier to understand for any audience.” [Researcher, country C]

In countries B and E, this new approach shifted communication from being at the end of the grant, to earlier in the research process, offering opportunities to obtain feedback from decision-makers to ensure that their data is relevant for policy development. In countries A and E, an early engagement with the government resulted in support for data collection, which helped with study progression and resolved setbacks experienced in the early stages of the project.

Perspectives varied in country D on the ideal timing for communication between key stakeholders. A researcher in country D preferred communication with decision-makers when the results were available as human resources were not available to support early communications. However, the MOH officer preferred discussions at the initial stages of the research process. The MOH officer from country D found value in early communications to support the coordination of the project results with internal departments at the MOH.

#### Context

##### Country size and cultural context

Costa Rica, being a small country with a solid health system, enabled easier facilitation of research adoption into policy and program changes. Both MOH and the researcher from Costa Rica noted that the lines of communication were clear and direct compared to larger countries. Having fewer people involved in the research and policy making arena allowed for timely action of outputs. Also, the Costa Rican population was described as a facilitator of change as they modeled a lifestyle of healthy eating habits. A researcher from Costa Rica noted that, “I think there’s an increased interest in health from the population...they are eating or interested in eating foods that are healthier.”

## Discussion

This is one of the first known studies to examine factors related to the uptake of sodium reduction research into policies and programs in LAC, a topic of interest given the high prevalence of hypertension and CVD, alongside high intakes of sodium in this population. A study conducted a retrospective policy analysis based on reviews of existing reports and semi-structured interviews in 4 LMIC to understand the factors that influenced the implementation of existing salt reduction interventions in each country [36]. The current study and the policy analysis identified similar factors that impact the research implementation of sodium reduction policies, such as strong leadership and support from the government and other international agencies, as well as adequate resources. The current study took a case study qualitative approach and uniquely capitalized on the context of a funded research consortium of 5 LAC to explore the barriers and facilitators to the integration of research into the policy process, considering the views of both researchers and MOH officers. This study demonstrated that partnerships between researchers and policy decision-makers can promote and/or impede the use of research in sodium reduction policies and programs. Leadership and support from the government, international non-governmental organizations such as PAHO, and the IDRC, as well as KT plans (e.g., integrated and/or end-of-grant) supported the mobilization of research information for use in policy and program development. The food industry’s conflicts and competing interests, and persuasive power over the government were observed as strong barriers to research adoption in nutrition policy and program development and changes. A policy mapping study conducted by PAHO [30] found that at least 16 countries in the Americas region have at least one dietary sodium reduction regulation in place that is in line with the “Best Buys” of WHO, which is the most cost-effective recommendations for the prevention and control of diet-related NCDs to reduce population sodium overconsumption [19]. Globally, countries have acted to reduce population sodium consumption using policies; however, these actions are not progressing fast enough to meet the global targets of WHO for addressing diet-related morbidity and mortality associated with NCD [19]. These policies include those focused on monitoring the sodium content of food supply, use of social marketing to design public health programs aiming to reduce sodium intake at the population level, and evaluation of health-economic variables, which are all areas of research funded under the IDRC consortium research project of interest in this study.

Leadership and support from the government are strong facilitators of changes in sodium reduction policies in LMIC [25, 26]. This study, and others, found that there is a strong likelihood that research evidence will be adopted by policy decision-makers if there is strong buy-in and support from the governing bodies [25, 26]. Almost all countries from the IDRC consortium had strong governmental support. Despite human resource challenges, governmental support led to the acquisition of high-quality data that, in some countries, was integrated into sodium reduction policies [28]. Other studies found that leadership and authority were key facilitators to the use of research in policymaking [25]. Evidence suggests that when MOH officers have close relationships with academic institutions, policy changes are more likely to occur, as documented in the Oceanic region for the implementation of palm oil, fruit, and vegetable policies [26]. These considerations have been documented with other public health policy topics, such as in Kenya, where strong political commitment and leadership was a key factor for the implementation of tobacco control policies [37].

Despite having partnerships with the government, the food industry has a strong influence over policy advocacy efforts, which has been widely cited as a barrier to nutrition policy development and change [38, 39]. This research confirmed that sodium reduction work is also met with lobbying efforts and resistance from the food industry. Some participants in this study averted questions related to the nature and type of resistance imposed by the food industry out of fear of negative repercussions. The food industry’s influence is a systemic issue and dates back as early as 1990 in the United Kingdom. In the United Kingdom, the food industry pressured the government to reject the recommended sodium intake of <2000 mg/day out of fear that the food industry would withdraw political funding [40]. Similarly, a Canadian study involving a document review of correspondences and exchanges related to Health Canada’s Healthy Eating Strategy observed a high number of interactions initiated by the food industry [39]. Of the documented exchanges, the food industry used a wide variety of tactics to influence Canada’s nutrition policy [39]. These are examples from high-income countries; however, we assume that the food industry’s pressure and influence are amplified in LMIC where resources are constrained, lack of competencies in technical knowledge, and political unpredictability are common [21].

Researchers and policymakers can utilize the food industry’s persuasive power over the government to their advantage, to sway public health nutrition efforts in their favor. This research found that it is possible to shift the food industry’s perception and attitude toward sodium reduction, as seen in Costa Rica. During the grant period, the Costa Rican government and the food industry renewed an alliance and commitment to further reduce sodium in food products based on the research generated from the IDRC-funded consortium research. This was possible as the researchers offered iterative feedback and communication with key stakeholders, where expectations were defined and concerns were addressed as they arose. This is similar to how the United Kingdom government was eventually able to work collaboratively with the food industry and used the influence of businesses to engage with consumers, by forming a partnership with them and other stakeholders to meet public health goals related to food, alcohol, health activity, and other health behaviors [41]. This progress further demonstrates that relationship management with multiple parties can advance public health goals, especially when each stakeholder has competing interests.

The leadership and commitment of PAHO/WHO to improve and protect the health of the Americas positively impacted research use in policymaking. The WHO/PAHO has a long-standing history of promoting and developing the capacity for the use of research data and evidence in policymaking and health systems [42]. The Evidence-Informed Policy Network (EVIPNet) program that PAHO/WHO adopted over a decade ago, has created positive impacts on this program and networks related to numerous public health policies [42]. Most importantly, the IDRC’s funding and support for sodium reduction research in LAC informed the WHO’s recent Global Sodium Benchmarks for Processed Foods. The data acquired from the IDRC research consortium was also used to develop new and revised sodium targets for PAHO that were released in October 2021 [32]. This was possible through the IDRC-funded consortium research and lessons learned from several countries that developed national and regional sodium reduction targets. The development of harmonized regional and global sodium reduction targets is a critical step to further strengthen country-level policies and programs and support member states in achieving a 30% reduction in mean population sodium intakes by 2025. It is important to note that this qualitative case study, design, and interpretations were independently developed and not driven by the IDRC. Rather the qualitative study was a separate study where the findings were used to supplement the program evaluation of the funded consortium and provided contextual information on the factors related to the uptake of research generated from the funded consortium into dietary sodium reduction policies.

The KT strategies developed and the high-quality research data generated had complementary effects that enabled researchers to generate discussions with policymakers and the food industry. Integrated and end-of-grant KT principles and strategies synthesize and disseminate research at various time points in the research cycle into audience-specific, relevant, and understandable information and tactics so that the evidence is more likely to be effectively adopted by end users [43]. In this study, KT capacity building among the funded countries was considered a major gain for future research. Overall, it has been reported that it takes an average of 17 years for evidence-based practices to be incorporated into general practice [44]. The research used to inform policies in LMIC could follow a similar lag-time pattern. The researchers developed their skillset and capacity on KT principles under this project, which included multiple training sessions, and country and project -specific KT workbooks that supported researchers in strategizing dissemination efforts. For a majority of the researchers in the IDRC project, KT was a new concept, but researchers quickly learned how to apply the principles. Future research initiatives in LMIC and LAC should embed integrated and end-of-grant KT activities to strategize the uptake of research findings in cultural and political contexts so that decision-makers can best use the research to support policy and program development and change.

The small sample size is a limitation of this study. Data saturation was not the focus of this qualitative inquiry as a case study approach was used, which has a greater level of flexibility than other qualitative approaches such as grounded theory or phenomenology. Rather, sample size determination was based on the bounds of the case that included all funded countries, a common practice in case study research. Additionally, the philosophical assumption can impact sample size. Positivism requires a large sample size to draw generalizable conclusions on a population of interest. In contrast, constructivism concerning case study methodology relies on a smaller sample to permit in-depth case-oriented analysis, where a case involving a single participant can provide in-depth insight into the topic of interest. This case involved 5 LAC who participated in a funded research consortium oriented in a constructivist lens, which has a focus on the historical and cultural context in which people live and work. The consortium members consisted of 5 country leads who led the research activities in their country under this grant. The 5 country leads are the research population of interest and were purposively sampled based on the unique role and contributions to the grant. There are no other eligible participants who could speak to the factors on the uptake of research evidence generated under the funded project into policies. In addition, MOH officers were selected based on their role in NCD development and implementation in the 5 funded countries, where incumbents occupying this role are limited or absent. As such, it was not feasible to recruit more than 5 researchers and 5 MOH officers from each country.

In addition to its originality, the strength of this study is the case study approach, which allowed for a level of flexibility with wide diversity in design, such as data collection through remote means [45]. Some may consider that a limitation of this work is the remote, online nature in which this was conducted; however, this is a feasible and economical alternative approach that is shown to produce similar findings to face-to-face interviews [46, 47]. The research team consisted of an English, Portuguese, and Spanish-speaking multi-disciplinary team from North, Central, and South America, which was a strength of this study [48]. The public health researchers of Central and South American origin were valuable contributors as they provided cultural understanding and different perspectives to this work. The inclusion of Spanish-speaking project team members further enhanced the trustworthiness and rigor of this cross-language research [48]. To enhance effective communications, Spanish-speaking team members should have been utilized to conduct the interviews in Spanish, the participant’s first language. A majority of the interviews were conducted in English as the preferred choice, and only a few interviews were conducted in Spanish. Despite this limiting factor, interview guides were translated into Spanish and sent to participants in advance of the interview. This allowed participants to prepare and have a better idea of the nature of questions to be asked, which is a practice that has been used in other qualitative studies [49].

## Conclusion

Researchers and policymakers are faced with several barriers and facilitators on how research is adopted into policies and programs for dietary sodium reduction. Although barriers and facilitators experienced in each country have cultural and political roots, the awareness and insights gained from this study will help guide countries in working through these issues in the future. Other LAC can extrapolate and customize the facilitators to meet their own country’s need to continue the efforts on policy nutrition and thus promote healthy eating and reduce CVD risks.

## Ethics approval and consent

Participants provided informed consent before the interview and for dissemination of case study findings. The Ontario Tech University Research Ethics Board reviewed this project and granted an ethics review exemption for the program evaluation component of the study (File # 14970) and reviewed the qualitative case study for ethical alignment with the Tri-Council Policy Statement 2 (File # 17292).

## Availability of data materials

The de-identified datasets (interview transcriptions, documents, and reflexive journal) used during the current study are available from the corresponding author on reasonable request pending the participant’s consent.

## Appendix A Supplementary data

Multimedia component1 Multimedia component1

Multimedia component2 Multimedia component2

Multimedia component3 Multimedia component3

Multimedia component4 Multimedia component4
