# Supplementary material for: Factors Impacting the Uptake of Research into Dietary Sodium Reduction Policies in Five Latin American Countries: A Qualitative Study
Source: Curr Dev Nutr. 2023 Apr 1;7(5):100073. doi: 10.1016/j.cdnut.2023.100073 (PMC10126926; doi:10.1016/j.cdnut.2023.100073)
Supplement: Multimedia component1 [file mmc1.docx]

**Supplementary Table 1. The funded countries sodium reduction policies during the grant period.**

| **Summary of Sodium Reduction Policies** | | **Funded Consortium Countries** | | | | |
| --- | --- | --- | --- | --- | --- | --- |
|  |  | **Argentina** | **Brazil** | **Costa Rica** | **Paraguay** | **Peru** |
| **Government policies** | NCD Policy | National | National | National | National | National |
|  | Specific policy for the reduction of salt/sodium intake | National | None | National | None | None |
| **Legislation** | Taxes to high salt/sodium foods | None | None | None | None | None |
|  | Regulation on marketing of foods high in salt | None | None | National^a^ | National^a^ | National |
|  | Nutritional labelling (include sodium content on nutritional panel) | National – Mandatory | National – Mandatory | National – Voluntary | National – Mandatory | None |
| **WHO “Best Buys”** | Reformulation of food products | National – Voluntary, Mandatory | National – Voluntary | National – Voluntary | National - Mandatory | None |
|  | Establishment of a supportive environment in public institutions | National | National | National | National | National |
|  | Behavior change communication and mass media campaign | National | None | Commitment from the government^b^ | Commitment from the government^b^ | Commitment from the government^b^ |
|  | Front-of-pack labelling | None | None | None | None | National^b^ |
| **Monitoring** | Population salt/sodium intake | National | National | National | National | None |
|  | Consumer knowledge, attitude and behavior | National | National | National | National | National |
|  | Salt/sodium content in foods – Reformulation | National | National | National | National | National**^b^** |

NCD = Non-communicable disease

^a^ A policy or program revised or updated over the funding period as captured in a program evaluation .

^b^ A policy or program newly developed over the funding period, as captured in a program evaluation
