# Supplementary material for: Factors Impacting the Uptake of Research into Dietary Sodium Reduction Policies in Five Latin American Countries: A Qualitative Study
Source: Curr Dev Nutr. 2023 Apr 1;7(5):100073. doi: 10.1016/j.cdnut.2023.100073 (PMC10126926; doi:10.1016/j.cdnut.2023.100073)
Supplement: Multimedia component2 [file mmc2.docx]

**Supplementary Table 2. Overview of barriers and facilitators to dietary sodium research uptake into policy in LAC**

| **Dimension(33)** | **Definition** | **Barriers and Facilitators** |
| --- | --- | --- |
| Actors | An individual or group who are directly or indirectly involved in the policy and program process. The governments, private organizations, non-governmental organizations, civil society, and academia are considered actors. | **Barriers**   - Food industry delayed policy development via lobbying and opposition due to commercial interests.   **Facilitators**   - Government political will to support the consortium objectives. - Research experts (regional and international) as influential partners. - Dual roles in research and policy making was a conduit to policy makers. - Non-governmental organizations provided technical guidance and knowledge exchange support. - International support provided consortium funding and technical guidance as well as supported mitigation strategies for risks. - Media influence draws attention to public health priorities to elicit policy changes. |
| Content | Attributes of the research data, which includes the relative advantage of the data. | **Facilitators**   - Research data generated under the IDRC project. - Qualitative research generated under the IDRC project. |
| Context | Social, economic, cultural factors and historical context. | **Barriers**   - Changes in government and shifts in public health priorities.   **Facilitators**   - Country size supported direct lines of communication to policy makers. - Cultural context modelled healthy lifestyle. |
| Process | Actions and outputs related to the policy process; communication channels used; dissemination of results; and resources. | **Barriers**   - Lack of human resources. - Communication challenges with policy makers.   **Facilitators**   - KT and implementation of research data. |

IDRC = International Development Research Centre

ram evaluation
