# Supplementary material for: Factors Impacting the Uptake of Research into Dietary Sodium Reduction Policies in Five Latin American Countries: A Qualitative Study
Source: Curr Dev Nutr. 2023 Apr 1;7(5):100073. doi: 10.1016/j.cdnut.2023.100073 (PMC10126926; doi:10.1016/j.cdnut.2023.100073)
Supplement: Multimedia component3 [file mmc3.docx]

**Manuscript ID:** CDN-D-22-00213

**Title:** Factors impacting the uptake of dietary sodium research into sodium reduction policies in five Latin American countries: A Qualitative Study

Janice Padilla-Moseley^1^, Bridve Sivakumar^1^, Nadia Flexner^2,3^, Ruben Grajeda^3^, Brenda Gamble^1^, Adriana Blanco Metzler^4^ and JoAnne Arcand^1^.

^1^Faculty of Health Sciences, Ontario Tech University, 2000 Simcoe Street North, Oshawa ON, L1G 0C5

^2^Department of Nutritional Sciences, University of Toronto, 1 King’s College Circle, Toronto, Ontario, Canada M5S 1A8

^3^Department of Non-Communicable Diseases and Mental Health, Pan American Health Organization; 525 23^rd^ St. NW, Washington, DC, United States of America, 20037

^4^Costa Rican Institute of Research and Teaching in Nutrition and Health (INCIENSA), Box 4-2250, Tres Ríos, Costa Rica Costa Rica

**Co-Authors**:

Bridve Sivakumar, email: [bridve.sivakumar@ontariotechu.net](mailto:bridve.sivakumar@ontariotechu.net); Nadia Flexner, email: [nadia.flexner@mail.utoronto.ca](mailto:nadia.flexner@mail.utoronto.ca); Ruben Grajeda, email: [Grajedar@outlook.com](mailto:Grajedar@outlook.com); Brenda Gamble, email: [Brenda.Gamble@ontariotechu.ca](mailto:Brenda.Gamble@ontariotechu.ca); Adriana Blanco-Metzler, email: [ablanco@inciensa.sa.cr](mailto:ablanco@inciensa.sa.cr); JoAnne Arcand, email: [JoAnne.Arcand@ontariotechu.ca](mailto:JoAnne.Arcand@ontariotechu.ca)

# **Appendix A** **–** Country lead interview script

1. Can you please tell me your position, title?
2. What is your involvement with policies, strategies and practices in your country?
3. How long have you been in this position?

Influence of IDRC research project on policies and program (e.g. program/practice changes) on sodium reduction

1. Please tell me about any other research projects you are aware of that focus on informing sodium reduction policies and programs in your country or in Latin America?
2. How does research knowledge influence your government’s likelihood of adopting a sodium reduction policy/program?

*Probe:*

1. Create local awareness.
2. Policy agenda setting, policy content and direction, evaluation of policy.
3. Can you think of other examples where research knowledge informed policies and programs in your government?
   1. Why do you think these policies were adopted?
   2. What inputs, in the form of key activities, and resources were required to implement the policy/program?
   3. Were all of these inputs and resources available?
4. In relation to the research conducted as part of this IDRC grant, please tell me about the kind of interactions you have had with policy makers during the grant period? (E.g., forums to hear about research findings, invitations to speak to policy makers, invitations to be active members in policy development).

*Probe:*

1. What did you learn from these exchanges with policy makers?
2. How do you feel about interacting with policymakers (knowledge users) about your research findings on sodium reduction and NCDs for policy development?
3. Can you think of time points in the research cycle where you could exchange information with policy makers? (E.g. idea generation, design, data collection, analysis, and application/dissemination of knowledge.)
4. When are appropriate time points to engage with policy makers to exchange information? (E.g. idea generation, design, data collection, analysis, and application/dissemination of knowledge.)

*Sodium focused policy and initiative changes from the onset of the project in 2016*

1. Based on a survey and mapping exercise led by the Pan American Health Organization (PAHO) on policies and initiatives focused on dietary sodium consumption in Latin American countries, the following policies and initiatives were found: [See country specific country profile].
2. Based on the country profiles, you have these policies and initiatives. Referring to the country profile, to your knowledge, what changes, if any, have occurred to this policy or program in your country since the start of the IDRC grant period in 2016?

*If change(s) occurred or new policy generated:*

1. How would you describe the change(s) to the new policies and programs?
2. In your opinion, to what extent did the IDRC research influence this policy or program?

*If no changes:*

1. What was the last known update or status of the policy or program?
2. Can you tell me why no changes have been made since 2016 (the start of the grant period)?

*Probe the factors involving:*

1. Maturity of the research results (e.g. too early to see outcomes).
2. Planning (e.g. project time lines or term in office).
3. Leadership (e.g. change in roles, champions to lead change, active involvement).
4. Resources (e.g. capacity, monitoring of adoption to ensure sustained changes, time challenges).
5. Priority (e.g., change in priority, focus on different policies).
6. Challenges in political environment (e.g. electoral uncertainty, policy instability, weak governance practices, violent conflict, humanitarian crisis).
7. In your opinion, what changes would you like to see with the policy or program?

If unsure of changes:

1. What was the last known update or status of the policy or program?
2. In your opinion, what changes would you like to see with the policy or program?
3. What are the proposed future plans for the policy or program, if any?

*Facilitators and barriers of implementing policies and programs in the IDRC countries.*

1. I am going to ask you about the barriers that impede the adoption of research into policies and programs. One type of barrier is **context,** which includes:

- social;
- economic and cultural factors;
- national and local policies;
- trends or governance; and
- historical context.

These contextual factors may influence policy development and implementation related to sodium reduction. Please describe any context barriers that would influence the adoption of sodium reduction policies and programs in your country.

*Probe the importance of*:

1. Challenges in political environment (e.g. electoral uncertainty, policy instability, weak governance practices, violent conflict, humanitarian crisis).
2. Changes in administration.
3. State structure and the relationship between the executive and legislative branches.
4. Restrictions in economic resources.
5. Now I would like to ask you about facilitators, which are factors that help or enable the uptake of research into policies and programs related to sodium reduction. What are **context facilitators** that have influenced the adoption research into policies and programs in your country?

*Probe:*

1. Organization’s attitudes and perceptions of research utilization.
2. Observing other Latin American countries adopt research data to create policies or laws to regulate sodium content in food sources.
3. Next, I would like to ask you about any **barriers** specifically related to the **content (or attributes) of the research data** on dietary sodium. For example, content of research data includes the:

- strength of the data;
- innovation of the research;
- gaps in the evidence, and
- relevance of the data to your country.

Please describe any barriers related to the content of research data that have influenced sodium reduction policies and programs in your country.

*Probes:*

1. Challenges with interpreting the research data.
2. Language too technical and statistical.
3. Value on NCD prevention is not clear.
4. Innovative findings.
5. Lack of credibility of research findings.
6. Now I would like to ask you about any **facilitators** related to **the content (or attributes) of the research data** that may influence the adoption into sodium reduction policies and programs as well as the relative advantage of the content. Please describe any facilitators related to the content of the research data that have influenced sodium reduction policies in your country.

*Probe:*

1. Type of research approach (e.g. biomedical or clinical research versus social science observational research).
2. Maturity of research in the field (e.g. empirical research, journals, conferences, presence of experienced researchers in the field),
3. Specificity of the research (e.g. targeted, short-term results, cost effective benefits).
4. Communication training on developing policy briefs, delivering testimonies to legislative bodies, executive branch members, or policymaking groups.
5. Information sources (e.g. media, emails, and brief publications).
6. I would like to ask you about **barriers** related to **policy actors** which are any individual or group who are directly or indirectly involved in the policy process. To name a few, policy actors may include:

- Governments;
- private organizations;
- non-governmental organizations;
- civil society; and
- academia.

Please describe any **barriers** related to the **actors** involved that would influence the creation of policies and programs related to dietary sodium.

*Probe:*

1. Research driven by the public sector versus academia.
2. Political culture (e.g. decision making based on experience and immediate pressures, rather than research evidence).
3. Technical background to understand and interpret research results.
4. Industry lobbying or interference.
5. Now, please describe any **facilitators** related to the **policy actors** involved that would influence the creation of policies and programs related to dietary sodium.

*Probe:*

1. Inter-professional social networks and collaborations. E.g. non-governmental organizations, official research organizations in the health sector
2. A dedicated facilitator or liaison (e.g. someone that is familiar with your country's political climate),
3. Mass media.
4. Key interest groups (E.g. members of the public, private industry).
5. International support (e.g. foreign donors).
6. I would like to ask you about any **process barriers** that may influence the creation of policies and programs on sodium reduction. Process factors are:

- actions and outputs related to the policy process;
- what policy actors do and why;
- communication channels used;
- dissemination of results; and
- resources that are used to promote or impede the use of research in policy.

Please describe any process barriers that may influence the creation of policies and programs on sodium reduction in your country.

*Probe:*

1. Complex process.
2. Resources (e.g. funding, adequate personnel, capable personnel, time constraints).
3. Poor communication channels between researchers (knowledge generators) and decision-makers (knowledge users).
   1. What changes can be made to promote optimal communication?
4. How does research results move (disseminate) within and across organizations?
5. Now, I would like to ask you about any **process facilitators** (e.g. actions and outcomes). Please describe any process facilitators that may influence the creation of policies and programs in your country.

*Probe:*

1. Communication channels (e.g. formal versus informal).
2. International support for research (e.g. financial support).
3. Advocacy efforts.
4. Policies and programs are one way to reduce dietary sodium at a population level. Tell me about any other types of sodium reduction interventions that you think would be helpful in reducing dietary sodium intake.
5. Do you have any further comments you would like to add before concluding our conversation today?

Thank you for your time.

## **Appendix B - Ministry of Health Officer interview script**

1. Can you please tell me your position, title?
2. What is your involvement with policies, strategies and practices in your country?
3. How long have you been in this position?

Influence of IDRC research project on policies and initiatives (e.g. program/practice changes) on sodium reduction

1. Please tell me about any other research projects that you are aware of which focus on informing sodium reduction policies and programs in your country or Latin American.
2. How does research knowledge influence your government’s likelihood of adopting a sodium reduction policy or program?

*Probe:*

1. Create local awareness.
2. Policy agenda setting, policy content and direction, evaluation of policy.
3. Can you think of other examples where research knowledge informed policies and programs in your government?
   1. Why do you think these policies were adopted?
   2. What inputs, in the form of key activities, and resources were required to implement the policy/program?
   3. Were all of these inputs and resources available?
4. What information sources are considered before your government adopts the information into policy and programs? E.g., peer review articles, systematic reviews, conferences.
5. In relation to the research conducted as part of this IDRC grant, or any other research project, please tell me about the kind of interactions you have had with researchers during the grant period, or for any other research project. (E.g., forums to hear about research findings, invitations to speak to researchers, invitations to be active members in research projects).

*Probe:*

1. What did you learn from these exchanges with researchers?
2. How do you feel about interacting with researchers about initiatives on sodium reduction and NCDs for policy development?
3. Can you think of time points in the policy cycle where you could exchange information with researchers? (E.g. needs assessment, policy identification, policy creation, stakeholder consultation, policy evaluation).
4. Based on the research outputs of the IDRC project [name a few outputs on meetings, publications and conferences], how do you think this information will be used in policy and program action going forward?
5. In your view, how could sodium reduction policies be prioritized to the top?

Sodium focused policy and initiative changes from the onset of the project in 2016

1. Based on a survey and mapping exercise led by the Pan American Health Organization (PAHO) on policies and initiatives focused on dietary sodium consumption in Latin American countries, the following policies and initiatives were found: [See Nadia’s country profile].
2. Based on the country profiles, you have these policies and initiatives in your country. Referring to the country profile, to your knowledge, what changes, if any, have occurred to this policy or program in your country since the start of the IDRC grant period in 2016?

*If change(s) occurred or new policy generated:*

1. How would you describe the change(s) or the new policy or program?
2. In your opinion, to what extent did the IDRC research, or any other research project, influence this policy or program?

*If no changes:*

1. What was the last known update or status of the policy or program?
2. Can you tell me which of the following factors may or may not have contributed to no changes have been made since 2016 (the start of the grant period)?

*Probe the factors involving:*

1. Maturity of the research results (e.g. too early to see outcomes).
2. Planning (e.g. project time lines or term in office).
3. Leadership (e.g. change in roles, champions to lead change, active involvement).
4. Resources (e.g. capacity, monitoring of adoption to ensure sustained changes, time challenges).
5. Priority (e.g., change in priority, focus on different policies).
6. Challenges in political environment (e.g. electoral uncertainty, policy instability, weak governance practices, violent conflict, humanitarian crisis).
7. Not aware of research outputs from this project.
8. In your opinion, what changes would you like to see with the policy or program?

*If unsure of changes:*

1. What was the last known update or status of the policy or program?
2. In your opinion, what changes would you like to see with the policy or program?
3. What are the proposed future plans for the policy or program, if any?

Facilitators and barriers of implementing policies and programs in the IDRC countries.

1. I am going to ask you about the barriers that impede the adoption of research into policies and programs. One type of barrier is **context,** which includes:

- social;
- economic and cultural factors;
- national and local policies;
- trends or governance; and
- historical context.

These contextual factors may influence policy development and implementation related to sodium reduction. Please describe any context barriers that would influence the adoption of sodium reduction policies and programs in your country.

*Probe the importance of*:

1. Challenges in political environment (e.g. electoral uncertainty, policy instability, weak governance practices, violent conflict, humanitarian crisis).
2. Changes in administration.
3. State structure and the relationship between the executive and legislative branches.
4. Restrictions in economic resources.
5. Key interest groups (e.g. members of the public, private industry).
6. Now I would like to ask you about facilitators, which are factors that help or enable the uptake of research into policies and programs related to sodium reduction. What are **context facilitators** that have influenced the adoption research into policies and programs in your country?

*Probe:*

1. Organization's attitudes and perceptions of research utilization.
2. Observing other Latin American countries adopt research data to create policies or laws to regulate sodium content in food sources.
3. Key interest groups (e.g. members of the public, private industry).
4. Next, I would like to ask you about any **barriers** specifically related to the **content (or attributes) of the research data** on dietary sodium. For example, content of research data includes the:

- strength of the data;
- innovation of the research;
- gaps in the evidence, and
- relevance of the data to your country.

Please describe any **barriers** related to the content of research data that may have negatively influenced sodium reduction policies and programs in your country.

*Probes:*

1. Challenges with interpreting the research data.
2. Language too technical and statistical.
3. Value on NCD prevention is not clear.
4. Innovative findings.
5. Lack of credibility of research findings.
6. Now I would like to ask you about any **facilitators** related to **the content (or attributes) of the research data** that may be helpful in influencing the adoption into sodium reduction policies and programs, as well as the relative advantage of the data. Please describe any facilitators related to the content of the research data that have influenced sodium reduction policies in your country.

*Probe:*

1. Type of research approach (e.g. biomedical or clinical research versus social science observational research).
2. Maturity of research in the field (e.g. empirical research, journals, conferences, presence of experienced researchers in the field),
3. Specificity of the research (e.g. timely, targeted and short-term results; cost effective benefits).
4. Information sources (e.g. media, emails, and brief publications).
5. Can you describe an example when research data drove political action in your country?

*Probe:*

1. What was the role of civil society in the uptake of research into political action? (E.g. engagement, support).
2. I would like to ask you about **barriers** related to **policy actors** which are any individual or group who are directly or indirectly involved in the policy process. To name a few, policy actors may include:

- Governments;
- private organizations;
- non-governmental organizations;
- civil society; and
- academia.

Please describe any **barriers** related to the **actors** involved that would negatively influence the creation of policies and programs related to dietary sodium.

*Probe:*

1. Research driven by the public sector, civil society and academia.
   1. How do you perceive these groups to work together in policy action?
2. Political culture (e.g. decision making based on experience and immediate pressures, rather than research evidence).
3. Technical background and capacity building in certain research areas (E.g. to understand and interpret research results).
4. Industry lobbying or interference.
5. Now, please describe any **facilitators** related to the **policy actors** involved that would be helpful in influencing the creation of policies and programs related to dietary sodium.

*Probe:*

1. Inter-professional social networks and collaborations (e.g. non-governmental organizations, official research organizations in the health sector).
2. A dedicated facilitator or liaison (e.g. someone that is familiar with your country's political and research climate).
3. Mass media.
4. Key interest groups (e.g. members of the public, private industry).
5. International support (e.g. foreign donors).
6. I would like to ask you about any **process barriers** that may negatively influence the creation of policies and programs on sodium reduction. Process factors are:

- actions and outputs related to the policy process;
- what policy actors do and why;
- communication channels used;
- dissemination of results; and
- resources that are used to promote or impede the use of research in policy.

Please describe any process barriers that may influence the creation of policies and programs on sodium reduction in your country.

*Probe:*

1. Complex process.
2. Resources (e.g. funding, adequate personnel, capable personnel, time constraints).
3. Poor communication channels between researchers (knowledge generators) and decision-makers (knowledge users). Do these channels need to be rebuilt?
4. How does research results move (e.g. disseminate) within and across your organization?
5. Now, I would like to ask you about any **process facilitators** (e.g. actions and outcomes). Please describe any process facilitators that may be helpful in influencing the creation of policies and programs in your country.

*Probe:*

1. Communication channels (e.g. formal versus informal).
2. International support for research (e.g. financial support).
3. Advocacy efforts.
4. Policies and programs are one way to reduce dietary sodium at a population level. Tell me about any other types of sodium reduction interventions that you think would be helpful in reducing dietary sodium intake.
5. Do you have any further comments you would like to add before concluding our conversation today?

Thank you for your time.
